# Litchi-Derived Polyphenol Alleviates Liver Steatosis and Gut Dysbiosis in Patients with Non-Alcoholic Fatty Liver Disease: A Randomized Double-Blinded, Placebo-Controlled Study

**DOI:** 10.3390/nu14142921

**Published:** 2022-07-16

**Authors:** Thananya Jinato, Maneerat Chayanupatkul, Thasinas Dissayabutra, Anuchit Chutaputti, Pisit Tangkijvanich, Natthaya Chuaypen

**Affiliations:** 1 Center of Excellence in Hepatitis and Liver Cancer, Department of Biochemistry, Faculty of Medicine, Chulalongkorn University, Bangkok 10330, Thailand; ji.thananya@gmail.com; 2 Alternative and Complementary Medicine for Gastrointestinal and Liver Diseases Research Unit, Department of Physiology, Faculty of Medicine, Chulalongkorn University, Bangkok 10330, Thailand; maneeratc@gmail.com; 3 Department of Biochemistry, Faculty of Medicine, Chulalongkorn University, Bangkok 10330, Thailand; thasinas@chula.md; 4 Department of Medicine, Phramongkutklao Hospital, Bangkok 10400, Thailand; anuchitwcog2002@hotmail.com

**Keywords:** NAFLD, metabolic syndrome, MRI-PDFF, polyphenol, Litchi chinensis-derived polyphenol, gut microbiota, *Akkermansia*

## Abstract

Preclinical data suggest the role of litchi extract in alleviating non-alcoholic fatty liver disease (NAFLD) by modulating gut microbiota. We aimed at investigating whether oligonol, a litchi-derived polyphenol, could improve liver steatosis and gut dysbiosis in patients with NAFLD. Adults with grade ≥2 steatosis, defined by an MRI proton density fat fraction (MRI-PDFF) of ≥11%, were randomly assigned to receive either oligonol or placebo for 24 weeks. The alteration in the MRI-PDFF and gut microbiota composition assessed by 16S ribosomal RNA sequencing were examined. There were 38 patients enrolled (*n* = 19 in each group). A significant reduction in the MRI-PDFF between week 0 and week 24 was observed in the oligonol group, while there was a non-significant decrease in the placebo group. A significant improvement in alpha-diversity was demonstrated in both of the groups. The oligonol-induced microbiota changes were characterized by reduced abundance of pathogenic bacteria, including *Dorea*, *Romboutsia*, *Erysipelotrichaceae UCG-003* and *Agathobacter*, as well as increased abundance of short-chain fatty acids (SCFAs)-producing bacteria, such as *Akkermansia*, *Lachnospira*, *Dialister* and *Faecalibacterium*. In summary, this study is the first to provide evidence that supports that oligonol improves steatosis through the modulation of gut bacterial composition. Our results also support the beneficial and complementary role of oligonol in treating NAFLD.

## 1. Introduction

Non-alcoholic fatty liver disease (NAFLD) represents one of the most common causes of chronic liver disease worldwide over the past two decades [1]. NAFLD is strongly linked to diabetes and obesity with its clinical spectrum ranging from steatosis to non-alcoholic steatohepatitis (NASH), progressive liver fibrosis, cirrhosis and, eventually, the development of hepatocellular carcinoma (HCC) [1]. It is estimated from a recent meta-analysis that the global prevalence of NAFLD is 28–36% and the overall prevalence in Asian populations is approximately 30%, with a rapidly increasing trend over time [2]. Moreover, it has become apparent that the clinical manifestations of NAFLD are beyond the liver, such as cardiovascular disease (CVD), chronic kidney disease (CKD) and extrahepatic malignancies [3]. Despite its high disease burden and potential implications in health, there is an absence of approved pharmacological therapies for patients with NAFLD. At present, the cornerstone of clinical management across the disease spectrum of NAFLD is lifestyle modifications, including exercise, diet and weight reduction [4,5]. Although the effectiveness of these interventions has been established, a sustained adherence to this approach might be difficult for most patients. Thus, additional complementary therapeutic options beyond conventional intervention are greatly needed. 

The pathophysiology of NAFLD is complex, which is associated with several factors, including metabolic syndrome, genetic susceptibility, lifestyle and environmental factors [6]. Additionally, an accumulating number of studies have indicated an essential role for the gut microbiota and its functional metabolites in the pathogenesis and progression of NAFLD [6]. An altered gut microbiota and microbial metabolites, including short-chain fatty acids (SCFAs), could accelerate steatosis and fibrosis through the gut–liver axis [7]. Recently, several approaches regarding the modification of the gut microbiota, to alleviate NAFLD and metabolic syndrome, were investigated in clinical and preclinical studies. In particular, the use of supplements of dietary natural products and functional foods have gained much attention, due to their health advantages with low toxicity profiles [8,9]. Among them, litchi (*Litchi chinensis* Sonn.), a subtropical fruit cultivated in Southeast Asia, has a potential to improve metabolic health, as it contains several beneficial substances with antioxidant, anti-inflammatory and tumor-preventive properties [10]. It was previously shown in alcohol-fed mice that the phenolic extract from litchi pulp could protect against liver injury by alleviating gut microbiota dysbiosis and intestinal barrier dysfunction [11]. Recent data also revealed that litchi seed extract could prevent obesity and altered the gut microbiota compositions in an animal model [12]. These preclinical results provide promising evidence supporting the use of litchi components as dietary supplements for the treatment of obesity and NAFLD by modulating gut dysbiosis. However, the beneficial role of this functional food in terms of modifying steatosis and altered gut microbiota has not been investigated in clinical studies. 

In this report, we aimed to determine whether oligonol, a commercially available oligomerized-polyphenol derived from litchi extract, could improve the severity of liver steatosis in patients with NAFLD. Additionally, we investigated the effect of oligonol supplementation on the microbial communities and SCFAs in these patients, thereby highlighting oligonol as a potential option that might alleviate NAFLD progression.

## 2. Materials and Methods

### 2.1. Participants and Study Design

A randomized, double-blinded, placebo-controlled study, recruiting patients with NAFLD at the King Chulalongkorn Memorial Hospital, Thailand, was conducted during July 2019 to June 2020. The study protocol was approved by the Institutional Review Board (IRB no. 242/62). The study was conducted in compliance with the Declaration of Helsinki and the principles of Good Clinical Practice. The informed consent was obtained from all of the participants. The study was also registered in the Thai Clinical Trial Registry (identification number: TCTR20200814001).

The inclusion criteria were patients aged 18 years and older. The diagnosis of NAFLD was performed by magnetic resonance imaging-derived proton density fat fraction (MRI-PDFF) with the presence of liver steatosis grade ≥2 (defined as MRI-PDFF ≥ 11%) [13]. Exclusion criteria included: (1) cirrhosis defined by clinical characteristics and/or radiological studies; (2) other liver diseases, such as concomitant chronic viral hepatitis B or C, autoimmune hepatitis and alcoholic liver disease; (3) HCC or other cancers; (4) CKD stage ≥3 (calculated glomerular filtration rate < 60 mL/min); (5) pregnancy and (6) previous history of litchi allergy. Patients were recommended not to receive any nutritional or herbal supplements, probiotics, prebiotics, antibiotics and proton pump inhibitors, as well as alcohol consumption for at least 3-month before the enrollment and throughout the study period. The basic demographic data and relevant clinical information were recorded, using a predefined questionnaire. In this study, individuals without any hepatic or systemic disease were also enrolled, as the healthy controls.

After completing the baseline assessment, patients with NAFLD were randomized in a 1:1 ratio to receive either oligonol (100 mg capsule; Amino Up Co. Ltd., Sapporo, Japan) or an identical-appearing placebo (contained 72% of microcrystalline cellulose, 23.5% of gelatin and 2.5% of sodium starch) twice a day for 24 weeks. The previous data in the healthy volunteers demonstrated that the recommended dose of oligonol (200 mg/day) did not result in any significant toxicity or adverse effects [14]. A stratified randomization was performed on the basis of patients’ age, gender and body mass index (BMI) by a computer-generated random number list. Moreover, both the investigators and participants were blinded to the study group. The participants were scheduled for interval visits at weeks 0 (baseline), 4, 12 and 24 (end of study). Clinical parameter evaluation (weight, height and waist circumference), laboratory tests and MRI-PDFF were assessed at weeks 0 and 24. In addition, the fecal sample specimens were collected at the same time points, to assess changes in gut microbiota composition. 

At week 24, the participants were requested to answer a questionnaire about their dietary habits during therapy (e.g., how often did they follow the recommended low-fat, low-carbohydrate diet? None, <50% of the time, or ≥50% of the time) and physical activity (e.g., how often did they exercise for at least 30 min during the study? Less than once a week, one–two times a week or ≥three times a week). The outcomes of the study were changes in the MRI-PDFF value at 24 weeks compared with baseline, including the proportions of patients with ≥10% and ≥30% relative changes in the MRI-PDFF at week 24. Additionally, the alteration of the gut microbiota composition, as well as other clinical and laboratory parameters at week 24, were assessed.

In this study, we calculated the sample size, based on the following formula [15]:

$$\mathrm{Standardized}\;\mathrm{difference}=\frac{\mathrm{Target}\;\mathrm{difference}}{\mathrm{Standard}\;\mathrm{diviation}},\;N=\frac{2}{d^{2}}\times \mathrm{Cp},\;\mathrm{power}\;$$

N is the number of participants in each group; d represents the standardized difference and C_p, power_ is a constant defined by the values chosen for the *p*-value and power. For the expected difference in MRI-PDFF of 30% based on a previous report [16] with the *p*-value of 0.05 and power of 80%, the calculated sample size in each group was approximately 19.

### 2.2. Serum Sample Collection and Measurement

To evaluate the clinical parameters, the fasting serum samples were analyzed by an automatic biochemistry analyzer (Alinity c System, Abbott Laboratories, Abbott Park, IL, USA) in the Central Laboratory of the King Chulalongkorn Memorial Hospital for alanine aminotransferase (ALT), aspartate aminotransferase (AST), total cholesterol (TC), triglycerides (TG), low-density lipoprotein (LDL), high-density lipoprotein (HDL), glucose and insulin. The Homeostatic Model Assessment for Insulin Resistance (HOMA-IR) was calculated, based on the following formula: HOMA-IR = (fasting glucose (nmol/L) × fasting insulin (µU/mL)/22.5). The blood specimens of the participants were obtained at week 0, prior to initiation of treatment. The serum samples were then separated by centrifugation and sent to the Central Laboratory on the same day.

### 2.3. Fecal Sample Collection and Microbial DNA Extraction and Sequencing

The participants in this study were instructed to follow the standard operating procedures (SOPs) for proper fecal collection, based on the International Human Microbiome Standard (IHMS) [17]. Approximately 1 g of fecal specimens were collected in the DNA/RNA Shield™- Fecal Collection tubes (Zymo Research Corp, Irvine, CA, USA) following vigorously shaken to stabilize the samples and stored at −80 °C until further analysis.

The total DNA extraction from fecal specimen was performed using the ZymoBIOMICS™ DNA Miniprep Kit (Zymo Research Corp, Irvine, CA, USA) according to the manufacturer’s protocol. DNA concentration and purity were measured by DeNovix™ UV-Vis spectrophotometer and stored at −20 °C until further analysis. Then, the amplicon-based 16S rRNA sequencing with V3-V4 hypervariable regions were amplified using the forward primers: 341F (5′-ACACTGACGACATGGTTCTACA-3′), and the reverse primers: 805R (5′- TACGGTAGCAGAGACTTGGTCT-3′) and sequenced by paired-end sequencing using the Illumina MiSeq 300 bp platform (Illumina, San Diego, CA, USA) at Génome Québec Innovation Centre (Montréal, QC, Canada). To obtain the clean data, the primer and adapter were trimmed, and the chimeric sequences were removed using Cutadapt [18]. The subsequent clean reads were clustered as amplicon sequence variants (ASVs), using DADA2 and annotated with the SILVA v.138.1 16S rRNA gene database [19]. The relative abundance and alpha-diversity were calculated, using the Phyloseq R package (v.1.38.0) (D2G Oncology, San Francisco Bay Area, CA, USA) [20]. The beta-diversity was calculated by the Bray–Curtis dissimilarity metric, and visualized by principal coordinate analysis (PCoA) plot, using MicrobiomeAnalyst web-based platform (https://www.microbiomeanalyst.ca/, accessed on 17 June 2022). 

### 2.4. Quantification of Butyryl-CoA: Acetate CoA-Transferase [BCoAT] Gene

To quantify the BCoAT and the V3-V4 16S gene expressions, qPCRs were performed, using 4X CAPITAL™ qPCR Green Master Mix (Biotech Rabbit). The BCoAT and V3-V4 16S genes were amplified by degenerated specific primers, as shown below [21,22]. The qPCR conditions started with a DNA-denaturation step at 95 °C for 15 min, followed by 40 cycles of denaturation at 95 °C for 15 s, annealing at a primer-specific temperature for 20 s, and extension at 72 °C for 30 s:

- BCoAT primer:
  Forward: 5′- GCIGAICATTTCACITGGAAYWSITGGCAYATG-3′
  Reverse: 5′- CCTGCCTTTGCAATRTCIACRAANGC-3′
- V3-V4 16S gene:
  Forward: 5′- CCTACGGGNGGCWGCAG-3′
  Reverse: 5′- CCTGCCTTTGCAATRTCIACRAANGC-3′

The cycle threshold (Ct) of each sample was compared to Ct of the standard curve. Then, the quantity of the BCoAT gene was calculated and normalized, using the V3-V4 gene (represents total bacteria). 

### 2.5. Statistical Analysis

The demographic data were analyzed, using SPSS (version 22.0.0, SPSS Inc., Chicago, IL, USA) and GraphPad Prism 8.0 (GraphPad Software Inc., San Diego, CA, USA). The category data were analyzed by chi-square and one-way ANOVA, while continuous data were analyzed using the Student’s *t*-test for parametric valuables, and the Mann–Whitney U test for nonparametric valuables. To test whether there were statistically significant differences in the relative abundance of the gut microbiota between the groups, a nonparametric Kruskal–Wallis test was performed. To test the relationship between the overall gut microbial community and clinical conditions, permutational multivariate analysis of variance (PERMANOVA) was performed, using the Bray–Curtis dissimilarity method. [23]. The comparison of changes in the relative abundance of the gut microbiota between post- and pre-treatment (symbolized by Δ) were calculated by non-parametric Mann–Whitney U test. An adjusted *p*-value < 0.05 was considered statistically significant.

## 3. Results

### 3.1. Clinical Parameters of Participants at Baseline and Week 24

A total of 38 participants with NAFLD (*n * = 19 in each group), and 15 healthy controls were included in the study (Table 1). Compared with the healthy controls, the patients with NAFLD had a higher proportion of male gender and higher AST and ALT levels. Among the patients with NAFLD, there was no significant difference between the oligonol and placebo groups, in terms of age, gender, body weight, waist circumference, body mass index (BMI), MRI-PDFF, AST, ALT and other baseline parameters. Of note, approximately 50% of the patients with NAFLD were overweight (BMI 23.0–27.5 kg/m^2^), and 50% were obese (BMI ≥ 27.5 kg/m^2^). 

**Figure 1:**
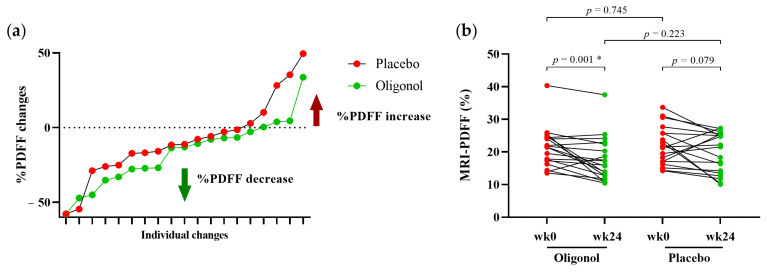
MRI-PDFF changes in the oligonol and placebo groups (**a**) linear graphs of percentage changes at week 24 from week 0; (**b**) MRI-PDFF values at week 0 and week 24 in each patient. * *p* < 0.05; MRI-PDFF, Magnetic Resonance Imaging Proton Density Fat Fraction; wk0, week 0; wk24, week 24.

Table 2 demonstrates the alterations in the clinical parameters between week 0 and week 24 in the oligonol and placebo groups. Our data showed that significant reductions in body weight, BMI, fasting blood sugar and ALT levels, as well as a trend of declined waist circumference, were observed in the oligonol group. However, only the reduction in body weight and BMI were found in the placebo group. Regarding the change in the MRI-PDFF between week 0 and week 24 in Figure 1, there was a significant decrease in the MRI-PDFF values demonstrated in the oligonol group (*p* = 0.001). A trend of decline without achieving significant change in MRI-PDFF was detected in the placebo group (*p* = 0.079). Despite these findings, however, the difference in MRI-PDFF between the two groups at week 24 did not reach statistical significance (*p* = 0.223). Proportions of the patients with ≥10% and ≥30% MRI-PDFF reduction at week 24 were achieved in 57.9% (11/19) and 26.3% (5/19), respectively, in the oligonol group, while the corresponding figures were observed in 52.6% (10/19) and 10.5% (2/19) in the placebo group. There was no significant difference between the two groups in terms of ≥10% and ≥30% MRI-PDFF reduction (*p* = 0.744 and *p* = 0.209, respectively). In contrast, the progressive steatosis ≥10% appeared in 5.3% (1/19) and 21.1% (4/19) of the individuals in the oligonol and placebo groups, respectively (*p* = 0.150).

Based on their self-reported questionnaire during the study, 55.6% (10/18) of the participants in the oligonol group indicated that they did not adhere to the recommended diet, while 88.9% (16/18) of the patients in the control group maintained dietary control (*p* = 0.018). Similarly, 44.4% (8/18) of patients in the oligonol group exercised less than once a week, while 77.8% (14/18) of the patients in the control group exercised at least one–two times a week (*p* = 0.329) (Table S1, Supplementary Materials).

### 3.2. Baseline of Gut Diversity and Composition

Based on 16S rRNA sequencing, an average of 49,189 ASVs per sample was obtained. After the quality filtering, denoising, read merging and chimera removal steps, we obtained an average of 21,761 ASVs per sample. (Table S2, Supplementary Materials). Finally, the rarefaction curve was plotted from the species richness, and all of the samples reached plateau curves. These results suggested that this sequencing depth could capture the species in all of the samples. 

**Figure 2:**
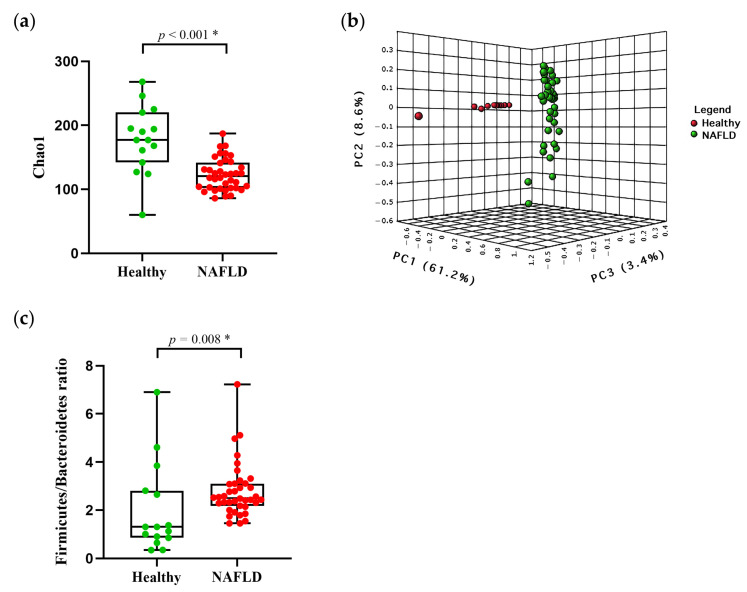
Gut bacterial diversities and *Firmicute/Bacteroidetes* ratio in healthy controls and patients with NAFLD at week 0 (**a**) Alpha diversity (Chao1); (**b**) Beta-diversity (Bray–Curtis distance); (**c**) *Firmicute/Bacteroidetes* ratio. * *p* < 0.05; NAFLD, Non-Alcoholic Fatty Liver Disease; PC, Principal Components.

The alpha diversity at baseline were analyzed, using Chao 1 and the Shannon index. In Figure 2a, Chao 1 showed a significant decrease in the patients with NAFLD (123.00 ± 24.72), compared to the healthy controls (178.30 ± 54.45, *p* = 0.001). The Shannon index was not significantly different between the patients with NAFLD and the healthy controls (*p* = 0.056) (Figure S1, Supplementary Materials). Additionally, the beta diversity of bacteria composition in the NAFLD group and the healthy controls was notably separated into two distinct enterotypes (PERMANOVA, *p* < 0.001), based on the Bray–Curtis distance, as shown in the PCoA plot (Figure 2b). 

To assess whether the difference in the bacterial composition of the gut microbiota was identified at baseline, we compared the most relative abundance of taxa in the healthy controls and patients with NAFLD. The dominant phyla in both of the groups were *Firmicutes*, *Bacteroidetes* and *Actinobacteria*, respectively (Figure S2, Supplementary Materials). However, the NAFLD group had a significantly higher abundance of *Firmicutes* compared with the healthy controls (62.13% vs. 50.28%, *p* = 0.006), but exhibited lower abundances of *Bacteroidetes* (24.25% vs. 39.13%, *p* = 0.008) and *Actinobacteria* (2.49% vs. 7.02%, *p* < 0.001; Figure S2, Supplementary Materials). Accordingly, the *Firmicutes/Bacteroidetes* ratio was significantly increased in the NAFLD group in comparison with the healthy controls (*p* = 0.008) (Figure 2c). 

We also detected the difference in the top 20 taxa at the family level between the two groups (Figure S3, Supplementary Materials). Among them, significantly a higher relative abundance of *Lachnospiraceae* (*p* < 0.001), *Acidaminococcaceae* (*p* = 0.011), *Coriobacteriaceae* (*p* < 0.001) and *Erysipelatoclostridiaceae* (*p* < 0.001) were found in the patients with NAFLD versus the healthy controls. In contrast, diminished abundances of *Bacteroidaceae* (*p* = 0.001) and *Sutterellaceae* (*p* = 0.042) were significantly observed in the patients with NAFLD compared with the healthy controls. However, the families *Ruminococcaceae*, *Prevotellaceae*, *Bifidobacteriaceae*, *Oscillospiraceae* and *Enterobacteriaceae* were comparable between the two groups.

**Figure 3:**
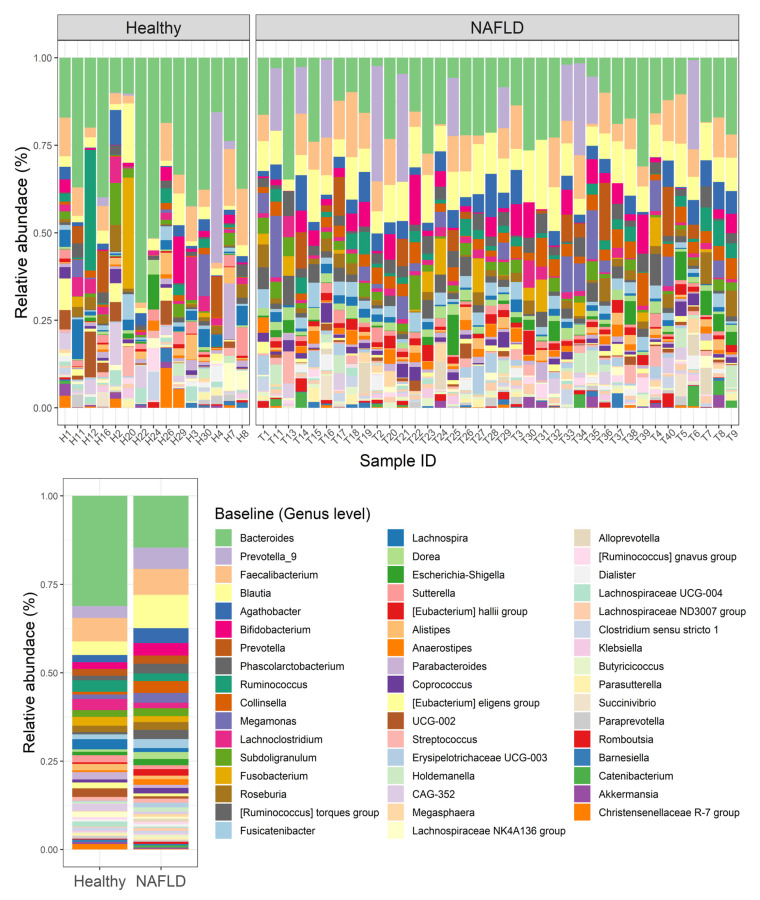
Top 50 relative abundance of bacterial genera in healthy controls and patients with NAFLD at week 0. NAFLD, Non-Alcoholic Fatty Liver Disease; UCG, uncultured genus-level group; CAG, Co-Abundance Groups.

Regarding the top 50 relative abundance of bacteria at the genus level, we also observed that there were 17 genera significantly different between the two groups (Figure 3). For instance, six genera, including *Bacteroides* (*p* < 0.001), *Lachnoclostridium* (*p* < 0.001), *Lachnospira* (*p* = 0.048), *Lachnospiraceae NK4A136* group (*p* = 0.017), *Lachnospiraceae UCG-004* (*p* = 0.015) and *Veillonella* (*p* = 0.002) were significantly lower in the patients with NAFLD compared with the healthy controls. In contrast, 11 genera, comprising *Blautia* (*p* < 0.001), *Agathobacter* (*p* = 0.011), *Phascolarctobacterium* (*p* = 0.014), *Collinsella* (*p* < 0.001), the *Ruminococcus torques* group (*p* < 0.001), *Fusicatenibacter* (*p* = 0.009), *Dorea* (*p* < 0.001), *Eubacterium hallii* group (*p* < 0.001), *Anaerostipes* (*p* = 0.002), *Lachnospiraceae ND3007* group (*p* = 0.002) and *Butyricicoccus* (*p* = 0.012) were enriched in the NAFLD group in comparison with the healthy controls.

### 3.3. Changes in Gut Diversities and Composition in the Oligonol and Placebo Groups at Week 24

**Figure 4:**
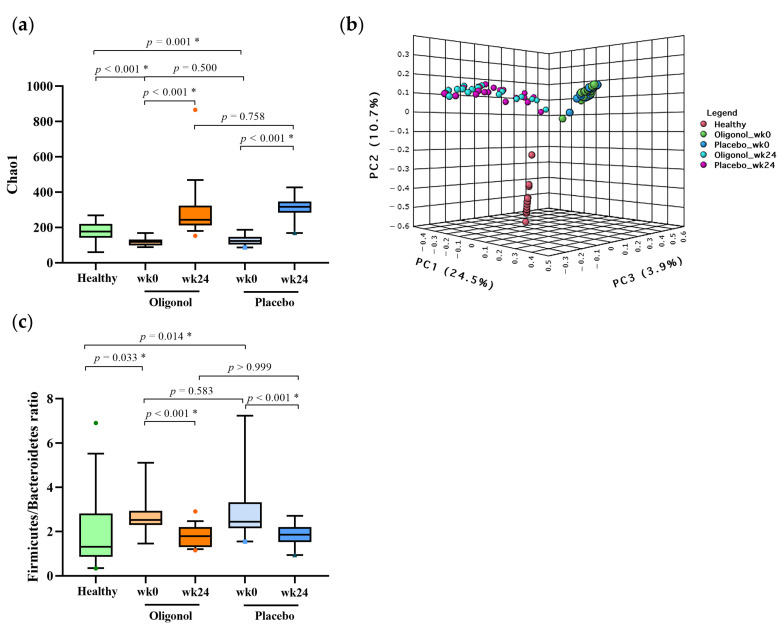
Gut diversities and *Firmicute/Bacteroidetes* ratio at week 0 and week 24 in the oligonol and placebo groups (**a**) Alpha diversity; (**b**) Beta-diversity (Bray–Curtis distance); (**c**) *Firmicute/Bacteroidetes* ratio. * *p* < 0.05; wk0, week 0; wk24, week 24; green dot, the outlier in healthy controls; oranges dot, the outlier in oligonol group; blues dot, the outlier in placebo group, PC, Principal Components.

We further examined whether gut diversities and composition could be modified after the supplementation. At baseline, there was no significant difference in the alpha diversity (Chao1), beta diversity and microbial community between the oligonol and placebo groups. At week 24, a significant improvement in the alpha-diversity was shown in both of the groups, when compared with their baseline data (*p* < 0.001) (Figure 4a). Similarly, the *Firmicutes/Bacteroidetes* ratio was significantly decreased at week 24 in both of the groups (*p* < 0.001) (Figure 4c). Regarding beta-diversity, significant changes in the clustering between weeks 0 and 24 were also observed (PERMANOVA, *p* < 0.001) (Figure 4b), indicating that the overall gut microbiota composition post-therapy was shifted from their baseline results in both of the groups. However, we did not observe any difference in the beta-diversity between the oligonol and placebo groups at the end of the study.

**Figure 5:**
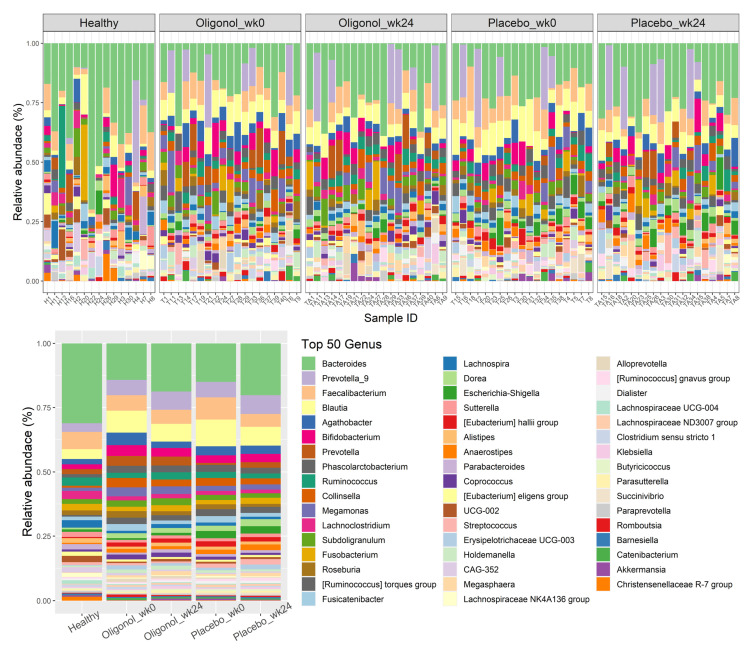
Top 50 relative abundance of bacterial genera at week 0 and week 24 in the oligonol and placebo groups.

To better understanding the changes in the specific gut microbiota composition after the supplementation, we compared the relative abundance of the overall top 50 genera before and after treatment. The differences in the genera identified between pre- and post-therapy in the oligonol and placebo groups are demonstrated in Figure 5. Remarkably, the oligonol group exhibited a significantly higher abundance of the genus *Lachnospira* and *Coprococcus* at week 24 compared with the placebo group (*p* = 0.049 and *p* = 0.040, respectively). In contrast, the genus *Dorea* was found to be significantly increased at the end of therapy in the placebo group compared with the oligonol group (*p* = 0.010).

To compare the effect of the oligonol and placebo, the relative abundance change in each of the groups was calculated, using the differences in microbial communities between post- and pre-treatment (Δ). At the phylum level, we did not find any significant difference between the groups. At the family level, we observed a decrease in the *Ruminococcaceae* in both of the groups; however, the family decreased more significantly in the placebo group than the oligonol group (*p* = 0.034). In contrast, the family *Erysipelatoclostridiaceae* was found to be significantly increased in the placebo group while it decreased in the oligonol group (*p* = 0.024) (Table S3, Supplementary Materials).

At the genus level, changes in eight genera were significantly identified between the oligonol and placebo groups (Table 3). Among these, four genera were observed to be enriched in relative abundance in the oligonol group, including Lachnospira (*p* = 0.047), Feacalibacterium (*p* = 0.019), Dialister (*p* = 0.044) and Akkermansia (*p* = 0.043). On the contrary, four of the genera had significantly higher abundance in the placebo group, including Dorea (*p* = 0.049), Agathobacter (*p* = 0.009), Erysipelotrichaceae UCG-003 (*p* = 0.011) and Romboutsia (*p* = 0.035). 

### 3.4. Quantification of BCoAT Genes at Week 0 and Week 24

**Figure 6:**
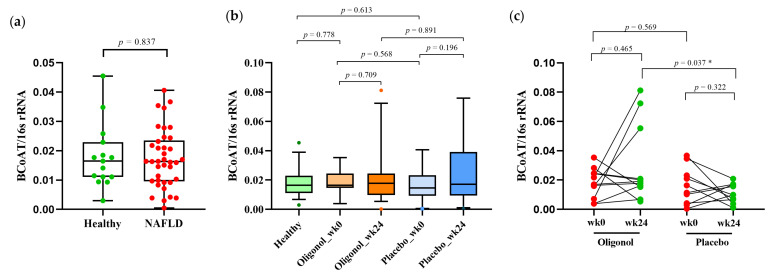
The levels of BCoAT gene in each group of participants (**a**) healthy controls and all patients with NAFLD at week 0; (**b**) the oligonol and placebo groups at week 0 and week 24; (**c**) only patients with decreased MRI-PDFF ≥10%. * *p* < 0.05; NAFLD, Non-Alcoholic Fatty Liver Disease; BCoAT, Butyryl-CoA: Acetate CoA-Transferase; 16 s rRNA, 16S ribosomal RNA; wk0, week 0; wk24, week 24.

The BCoAT gene was used to represent the butyrate level in fecal samples. At baseline, our data showed that the BCoAT levels were similar between the fecal specimens obtained from the healthy controls and the patients with NAFLD (Figure 6a). At week 24, the BCoAT levels did not differ significantly from baseline in both the oligonol and placebo groups (Figure 6b). However, in a subgroup analysis including only the patients whose fat levels decreased ≥10% at week 24, the BCoAT levels were significantly higher in the oligonol group when compared with the placebo group (Figure 6c). The information about the individual patients with fat levels that had decreased ≥10% is shown in Table S4 (Supplementary Materials).

## 4. Discussion

Oligonol is a litchi-derived polyphenol, comprised of catechin-type monomers and low-molecular-weight oligomers [10]. It has been shown, in in vitro and in vivo studies, that oligonol exhibits favorable and positive effects on health promotion, such as anti-oxidative stress, anti-obesity and anti-cancer [10]. However, the role of oligonol on the improvement of liver steatosis and in modifying gut microbiota have not been investigated in patients with NAFLD. In this study, we reported that oligonol was able to improve steatosis, as determined by MRI-PDFF. Moreover, there were significantly declines in other clinical and laboratory parameters, such as fasting glucose, liver biochemistry tests and BMI, as well as a trend of a decline in waist circumference. In the placebo group, however, decreasing BMI was only observed at week 24. Additionally, the oligonol therapy was associated with an improvement in gut dysbiosis and BCoAT levels, particularly among the patients who had significantly improved steatosis. Together, these results might suggest the pathological role of gut dysbiosis in the development of NAFLD, which could partially improve by an oligonol supplement.

At present, liver biopsy is the gold standard for the assessment of liver histopathology in patients with NAFLD. However, this invasive technique might be associated with procedure-related complications; thus, it is not practical for quantifying the dynamic changes of steatosis [24]. Among non-invasive methods, the MRI-PDFF is the most reliable alternative to liver biopsy in detecting steatosis and has been used as a primary endpoint in several clinical trials, because of its feasibility and accuracy [25]. In this study, we found a significant decline in the MRI-PDFF at the end of therapy only in the oligonol group, but did not observe such a change in the placebo group. Emerging data also indicate that ≥30% relative changes in MRI-PDFF is associated with a significant histological improvement and could be used as a surrogate marker for assessing treatment outcome [26]. Similarly, a threshold of MRI-PDFF relative reduction of ≥10% could be used as an appropriate alternative of ≥1-grade reduction in steatosis with a high sensitivity [27]. Together, we applied the cut-off ≥30% and ≥10% relative changes in the MRI-PDFF values as the outcomes for dynamic changes in steatosis at week 24. In this report, almost 30% of the oligonol group and 10% of the control group achieved steatosis ≥3 0% MRI-PDFF reduction. Additionally, approximately 58% and 53% in the corresponding groups had an MRI-PDFF improvement ≥10%. In contrast, progressive steatosis ≥10% appeared in approximately 5% and 21% of the participants in the respective groups. 

A better improvement of steatosis by oligonol might be related to several mechanisms, including its anti-inflammatory effects and its regulation of lipid metabolism. In a mouse model of type 2 diabetes, for example, it was shown that oligonol was able to attenuate liver injury through diminished oxidative stress and lower hepatic lipids via the downregulation of the sterol regulatory element-binding protein-1 [28]. In this study, it should be noted that the participants in the oligonol group had a significant decline in BMI, despite relatively less exercise and adherence to the recommended diet in comparison with those of the control group. These results might indicate that the oligonol has a direct modulatory effect on weight reduction. With respect to the anti-obesity effect, it was shown in preclinical experiments, indicating that oligonol enhanced the lipolysis and effectively inhibited the adipogenesis by repressing several major adipogenic genes [29,30]. In line with our report, a previous randomized, double-blinded, placebo-controlled study conducted in Japan demonstrated that a 10-week supplement of oligonol promoted weight loss and ameliorated metabolic syndrome by diminishing visceral fat and insulin resistance [31]. 

Apart from conventional metabolic-related mechanisms, accumulating evidence has indicated that gut dysbiosis plays a crucial role in the pathogenesis of NAFLD [6]. Indeed, several animal studies of NAFLD have described the changes in the gut microbiota signatures. Compared with healthy individuals, increasing evidence has also suggested that an altered gut microbiota composition is distinctly detected in patients with NAFLD at the level of phylum, family and genus [32,33]. Based on available data, however, there is heterogeneity across the studies [32,33]. In this study, our data clearly demonstrated that patients with NAFLD had a reduced diversity of bacteria compared with healthy individuals, and also displayed a distinct signature of their gut microbiota. The patients with NAFLD exhibited a higher abundance of the phylum *Firmicutes*, but a lower rate of *Bacteroidetes* compared to the control group. As a result, an increased *Firmicutes/Bacteroidetes* ratio was observed in the patients with NAFLD, which was in line with previous reports [33,34]. However, such an elevated ratio might be different and not yield consistent results in all of the reports [35]. Our findings also showed that the patients with NAFLD harbored an increase in the genera *Escherichia*, *Streptococcus*, *Dorea*, *Shigella*, *Roseburia* and *Ruminococcus*, as well as significantly decreased *Alistipes* and *Oscillibacter*, which were comparable to prior data [32,33,36]. In contrast, there were some discrepancies found between our study and previous reports, which included the genera *Coprococcus*, *Eubacterium*, *Faecalibacterium* and *Prevotella.*

The supplementation with several dietary polyphenols has been shown to modify gut microbiota and thus alleviate NAFLD [8,9]. In particular, previous reports in animal studies revealed that litchi-derived products exerted beneficial functions by modulating the gut microbiota composition. For instance, a previous study of alcohol-fed mice demonstrated that the litchi extract had a hepatoprotective effect that regulated by improving the microbiota dysbiosis and intestinal barrier dysfunction [11]. Additionally, a supplement of litchi pulp phenolics led to an increased abundance of the beneficial bacteria (e.g., *Akkermansia*, *Lactobacillus*, *Coprococcus* and *Bacteroides uniformis*) and a lower abundance of harmful bacteria (*Enterococcus and Aggregatibacter*) in dextran sulfate sodium-induced colitis [37]. Moreover, a recent report indicated that litchi seed extract effectively improved obesity, insulin resistance, glucose metabolism and the intestinal barrier in animal models using a high-fat diet [12]. Litchi seed extract could also alter the gut microbiota composition through an increased proportion of SCFA-producing bacteria, such as *Cetobacterium*, *Trichococcus*, *Aeromonas* and *Staphylococcus* [12]. Despite the observations in pre-clinical studies, the putative effects of litchi-derived products in modulating gut microbiota might not directly translate into humans. Thus, clinical studies regarding the relationship between oligonol, liver steatosis and gut microbiota are needed.

In the current study, we demonstrated that oligonol supplementation was able to restore gut microbiota diversity and composition. Specifically, we observed a significant improvement in the alpha-diversity in patients with NAFLD who received oligonol. Additionally, the beta-diversity representing the community structure of gut microbiota revealed a significant shift away from baseline, but was dissimilar to that observed in the healthy controls. These findings might indicate the partial restoration of the microbial balance and gut health following supplementation with oligonol. Of note, the improvement in the alpha-and beta-diversities was also similarly observed in the placebo group. This explanation of these findings was not clear but might indicate a relatively better adherence to the lifestyle change recommendations in the control group, as documented by their self-report questionnaires. In agreement with our findings, it has been demonstrated that combining physical activity and nutritional modulations may potentially restore microbial balance and improve gut health [38].

Although there was no significant change in the alpha- and beta-diversities between the oligonol and placebo groups, our results revealed an altered microbial composition that differentiated between the studied groups. Specifically, the oligonol therapy was associated with a lessening abundance of the pathogenic bacteria and an increased enrichment of the beneficial bacteria compared to the placebo. At the end of the therapy, the oligonol treatment significantly reduced the abundance of *Dorea*, *Romboutsia*, *Erysipelotrichaceae UCG-003* and *Agathobacter* compared to the placebo. The genera *Dorea* was shown to be elevated in pediatric NAFLD and was identified as the gut microbiota signature of progressive steatosis to NASH [39]. It was also demonstrated in patients with NAFLD that a high abundance of *Dorea* was correlated with elevated levels of volatile organic compounds (VOCs) [39]. Such potentially toxic VOCs could contribute to liver inflammation and apoptosis, leading to progressive liver disease, as recently postulated in an animal model [40]. Regarding *Romboutsia*, it was found as a principal genus associated with the the severity of NAFLD and diabetes [41]. Notably, oligonol therapy was also associated with a significant reduction in *Erysipelotrichaceae*. An increased abundance of *Erysipelotrichaceae* was shown to be correlated with an increased gut inflammation that was linked to colorectal cancer [42]. Moreover, a higher *Erysipelotrichaceae* level was observed in obese individuals and directly associated with progressive steatosis [43]. Thus, our results demonstrated that oligonol supplementation could improve gut health in the patients with NAFLD by decreasing the pathogenic bacteria.

The gut microbiota composition also regulates the abundance of SCFAs, the main metabolite products generated by bacteria, principally including acetate, propionate and butyrate. The SCFAs perform various physiological functions, such as promoting intestinal epithelium proliferation and enhancing gut barrier integrity [44]. Moreover, SCFAs have the ability to modulate energy regulation, immune activity and inflammatory response [44]. It has been shown in both animal and human studies that an abundance of SCFAs-producing bacteria is negatively correlated with liver inflammation and steatosis [45]. Moreover, alterations in the SCFAs levels produced by the gut microbiota have contributed to developing obesity in various animal studies, indicating that the administration of SCFAs might reduce or alleviate weight gain [46]. Thus, targeting gut dysbiosis with dietary supplements leading to an increase in SCFA production may be a promising strategy in the patients with NALFD and the overweight. In this study, several major SCFAs-producing bacteria were significantly decreased in the patients with NAFLD. Following oligonol therapy, we found that *Akkermansia*, *Lachnospira*, *Faecalibacterium* and *Dialister* were significantly enriched compared to those of the placebo group. In agreement with the increased abundance of the SCFAs-producing bacteria, we also demonstrated elevated levels of the fecal BCoAT/16s rRNA gene, a reliable semiquantitative assay for butyrate [21] in the oligonol group, compared to the placebo group, mainly in the subgroup of the individuals who had reduced MRI-PDFF values ≥ 10%.

Among them, *Faecalibacterium* is a main butyrate producer that plays an essential role in gut physiology and has been recognized as a biomarker of gut health [47]. The bacterium has been reported to regulate hepatic fat content and lipid compositions, as well as decreasing adipose tissue inflammation in animal studies [47]. In patients with NAFLD, the depletion of *Faecalibacterium* was a remarkable characteristic in a large BMI- and sex-matched population study [48] and was also positively correlated with the severity of liver fibrosis in a recent report [49]. Regarding *Akkermansia*, this mucin-degrading bacterium has been consistently linked to a protective effect against obesity and metabolic-associated diseases, including type 2 diabetes and NAFLD [50]. Apart from its valuable function in maintaining the gut epithelial integrity, *Akkermansia* is capable of promoting the growth of other beneficial bacteria and has been increasingly recognized as a promising candidate for the next generation of probiotics [51]. Interestingly, it was recently shown in an animal model that *Akkermansia* was able to prevent NAFLD development by modulating the gene expression that regulates fat synthesis and liver inflammation [52]. In a double-blinded, placebo-controlled study, it was also demonstrated that the administration of oral *Akkermansia* could alleviate metabolic syndrome in overweight or obese individuals [53]. Together, these data might indicate that the beneficial role of oligonol is related to its effect on the reduction in gut dysbiosis and, thus, improving metabolic disorders and diminishing liver steatosis.

Our data have some strengths. This is the first study in humans investigating the effect of litchi-derived polyphenol on steatosis and gut microbiota in NAFLD. In addition, we applied sequential MRI-PDFF, which is now regard as the most reliable non-invasive method for the measurement of liver steatosis [25]. This technique appears better suited for the assessment and follow-up of patients to quantify the changes in steatosis after the intervention, whereas conventional ultrasound or controlled attenuation parameter (CAP) has less accuracy. Despite these advantages, the study had some limitations. First, we included a relatively small number of participants in each group of the study. Second, the self-report information on the lifestyle interventions in terms of dietary behavior and physical activity might not be fully comprehensive. Additionally, the placebo group displayed a better adherence to the dietary control and a trend towards more physical activity in comparison with the oligonol group. These limitations highlight the complexity and interaction of several factors influencing steatosis and gut dysbiosis in patients with NAFLD.

## 5. Conclusions

This study is the first to provide evidence that the beneficial effects of oligonol on the alleviation of steatosis in patients with NAFLD could occur through the modulation of gut microbiota and its metabolites. These results recommend the complementary role of oligonol in treating NAFLD beyond lifestyle modification. Additional randomized, double-blind, placebo-controlled studies with larger sample sizes, as well as a longer duration, are needed to verify our interesting observations.

## Figures and Tables

**Table 1:** Baseline characteristics of all participants in this study.

	Healthy (*n* = 15)	Oligonol (*n* = 19)	Placebo (*n* = 19)	*p*-Value
Age (years)	50.7 ± 7.9	50.8 ± 10.1	49.5 ± 13.8	0.993
Male gender, *n* (%)	3(20%)	12 (63.2%)	11 (57.9%)	0.012 *^a^,0.026 *^b^,0.740 ^c^
BMI (kg/m^2^)	23.4 ± 2.3	27.9 ± 2.6	27.9 ± 3.3	<0.001 *^a,b^, 0.968 ^c^
Waist circumference (cm)		95.9 ± 6.8	95.1 ± 7.1	0.728
MetS, No *n* (%)		4 (21.1%)	3 (15.8%)	0.676
AST (IU/L)	20.3 ± 7.9	25.9 ± 5.1	34.4 ± 17.3	0.014 *^a^, 0.001 *^b^, 0.059 ^c^
ALT (IU/L)	17.3 ± 8.4	42.8 ± 17.5	53.6 ± 26.0	<0.001 *^a,b^, 0.280 ^c^
Fasting blood sugar (mg/dL)		111.0 ± 41.4	96.5 ± 17.4	0.293
Total cholesterol (mg/dL)		192.0 ± 31.7	190.1 ± 36.8	0.737
Triglyceride (mg/dL)		140.1 ± 70.8	133.3 ± 76.4	0.737
HDL (mg/dL)		50.0 ± 11.8	46.5 ± 11.1	0.357
LDL (mg/dL)		119.9 ± 27.1	126.2 ± 31.6	0.516
HOMA-IR (median ± IQR)		2.6 ± 3.0	2.4 ± 0.9	0.293
MRI-PDFF (%)		21.4 ± 6.1	22.0 ± 5.9	0.378

Data as shown in mean ± SD; *n* (%); * *p* <0 .05; ^a^ = Healthy vs. Oligonol; ^b^ = Healthy vs. Placebo; ^c^ = Oligonol vs. Placebo; BMI, body mass index; MetS, metabolic syndrome; AST, aspartate transaminase; ALT, alanine aminotransferase; HDL, high-density lipoprotein; LDL, low-density lipoprotein; HOMA-IR, Homeostatic model assessment of insulin resistance; MRI-PDFF, Magnetic Resonance Imaging Proton Density Fat Fraction.

**Table 2:** Clinical data of patients with NAFLD in the oligonol and placebo groups at week 0 and week 24.

	Oligonol (*n* = 19)			Placebo (*n* = 19)			*p*-Value ^ϯ^
	Week 0	Week 24	*p*-Value	Week 0	Week 24	*p*-Value	
Body weight (kg)	78.1 ± 9.7	76.6 ± 10.1	0.004 *	76.3 ± 13.0	75.2 ± 12.4	0.024 *	0.697
Waist circumference (cm)	95.9 ± 6.8	94.4 ± 6.8	0.074	95.1 ± 7.1	93.7 ± 6.9	0.089	0.761
BMI (kg/m^2^)	27.9 ± 2.6	27.3 ± 2.6	0.007 *	27.9 ± 3.3	27.5 ± 3.3	0.027 *	0.942
AST (IU/L)	25.9 ± 5.1	25.4 ± 7.1	0.569	34.4 ± 17.3	31.1 ± 11.8	0.294	0.065
ALT (IU/L)	42.8 ± 17.5	37.5 ± 14.0	0.036 *	53.6 ± 26.0	46.7 ± 23.5	0.144	0.422
Total bilirubin (mg/dL)	0.6 ± 0.2	0.6 ± 0.3	0.494	0.7 ± 0.3	0.9 ± 0.3	0.006 *	0.002 *
Fasting blood sugar (mg/dL)	111.0 ± 41.4	102.4 ± 33.5	0.005 *	96.5 ± 17.4	93.8 ± 12.2	0.163	0.919
Total cholesterol (mg/dL)	192.0 ± 31.7	194.3 ± 42.7	0.499	190.1 ± 36.8	185.0 ± 35.8	0.251	0.549
Triglyceride (mg/dL, median ± IQR)	121.0 ± 90.0	146.0 ± 121.0	0.026 *	108.0 ± 38.0	149.0 ± 90.0	0.020 *	0.770
HDL (mg/dL)	50.0 ± 11.8	48.5 ± 10.6	0.137	46.5 ± 11.1	44.7 ± 9.6	0.080 *	0.219
LDL (mg/dL)	119.9 ± 27.1	116.4 ± 29.2	0.446	126.2 ± 31.6	122.3 ± 36.4	0.365	0.582
HOMA-IR (median ± IQR)	2.6 ± 3.0	3.1 ± 2.2	0.546	2.4 ± 0.9	3.1 ± 2.6	0.010 *	0.804
MRI-PDFF (%)	21.4 ± 6.1	17.6 ± 6.8	0.030 *	22.0 ± 5.9	19.7 ± 6.2	0.077	0.215

Data as shown in mean ± SD; median ± IQR; * *p* <0.05; BMI, body mass index; MetS, metabolic syndrome; AST, aspartate transaminase; ALT, alanine aminotransferase; HDL, high-density lipoprotein; LDL, low-density lipoprotein; HOMA-IR, Homeostatic model assessment of insulin resistance; MRI-PDFF, Magnetic Resonance Imaging Proton Density Fat Fraction, ^†^ Comparison between the oligonol and placebo groups at week 24.

**Table 3:** Post- and pre-treatment significant changes (Δ) of relative abundance at the genus level in the oligonol and placebo groups.

**Enriched in the Oligonol Group**	**ΔOligonol**		**ΔPlacebo**		***p*−Value**
	**Mean**	**SD**	**Mean**	**SD**	
g_*Lachnospira*	0.005	0.011	−0.002	0.009	0.047
g_*Feacalibacterium*	−0.005	0.015	−0.032	0.043	0.019
g_*Dialister*	0.002	0.005	−0.002	0.006	0.044
g_*Akkermansia*	0.005	0.016	−0.004	0.011	0.043
**Enriched in the Placebo Group**	**ΔOligonol**		**ΔPlacebo**		***p*−Value**
	**Mean**	**SD**	**Mean**	**SD**	
g_*Dorea*	−0.001	0.011	0.007	0.011	0.049
g_*Agathobacter*	−0.022	0.030	−0.002	0.017	0.009
g_*Erysipelotrichaceae UCG−003*	−0.006	0.013	0.009	0.017	0.011
g_*Romboutsia*	−0.005	0.009	0.002	0.009	0.035

Δ, Changes in the relative abundance of the gut microbiota between post- and pre-treatment; UCG, uncultured genus-level group.

## Data Availability

Data available in the Sequence Read Archive (SRA) submission number SUB11619249 (BioProject ID: PRJNA849917).

## Supplementary Materials

The following supporting information can be downloaded at: https://www.mdpi.com/article/10.3390/nu14142921/s1, Figure S1: Baseline Shannon’s diversity in patients with NAFLD and healthy controls; Figure S2: Relative abundance at phylum level in patients with NAFLD and healthy controls; Figure S3: Relative abundance of top 20 families in patients with NAFLD and healthy controls; Table S1: Lifestyle modification of the participants; Table S2: Read summary; Table S3: Post- and pre-treatment changes (Δ) of relative abundance at the family level in the oligonol and placebo groups; Table S4: Patients with percent MRI-PDFF decreased ≥10% at week 24.
