# Supplementary material for: Litchi-Derived Polyphenol Alleviates Liver Steatosis and Gut Dysbiosis in Patients with Non-Alcoholic Fatty Liver Disease: A Randomized Double-Blinded, Placebo-Controlled Study
Source: Nutrients. 2022 Jul 16;14(14):2921. doi: 10.3390/nu14142921 (PMC9319370; doi:10.3390/nu14142921)
Supplement: Supplementary file 1 [file nutrients-14-02921-s001.zip › Supplementary Materials of Figures.pdf]

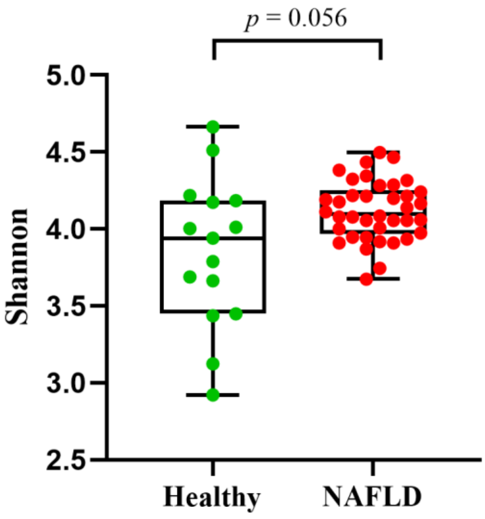

Figure S1. Baseline Shannon's diversity in patients with NAFLD and healthy controls.

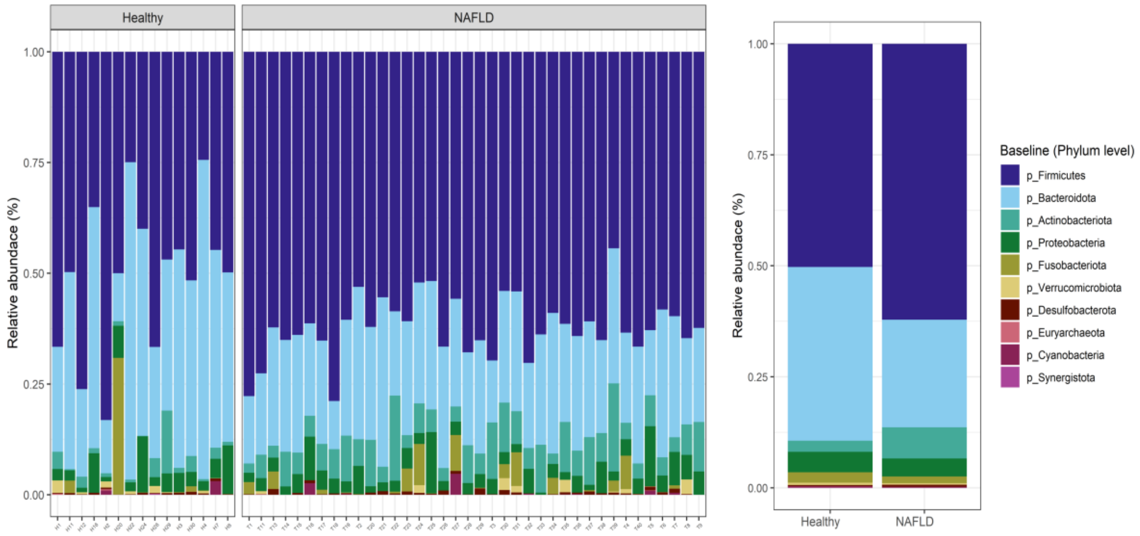

Figure S2. Relative abundance at phylum level in patients with NAFLD and healthy controls.

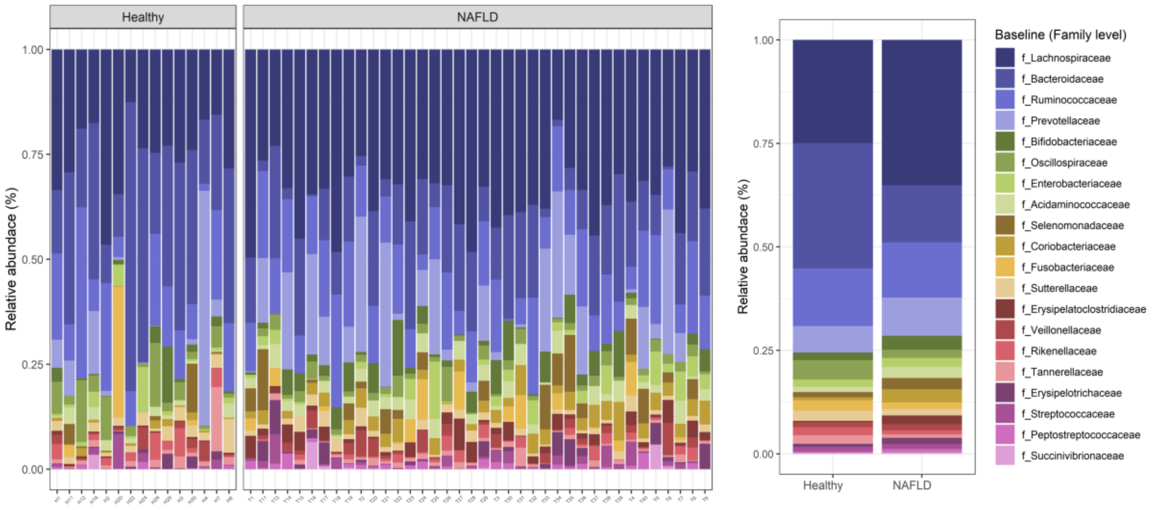

Figure S3. Relative abundance of top 20 families in patients with NAFLD and healthy controls.
