# Supplementary material for: Litchi-Derived Polyphenol Alleviates Liver Steatosis and Gut Dysbiosis in Patients with Non-Alcoholic Fatty Liver Disease: A Randomized Double-Blinded, Placebo-Controlled Study
Source: Nutrients. 2022 Jul 16;14(14):2921. doi: 10.3390/nu14142921 (PMC9319370; doi:10.3390/nu14142921)
Supplement: Supplementary file 1 [file nutrients-14-02921-s001.zip › Supplementary Materials of Tables.pdf]

Table S1 Lifestyle modification of the participants

Physical activity	Group		Total	*p*–Value
	Oligonol	Placebo		
less than once per week	8 (44.4%)	4 (22.2%)	12 (33.3%)	0.329
1-2 times per week	8 (44.4%)	10 (55.6%)	18 (50%)	
more than 3 times per week	2 (11.1%)	4 (22.2%)	6 (16.7%)	
Total	18 (100%)	18 (100%)	18 (100%)	
**Diet control**	**Group**		**Total**	***p*–Value**
	Oligonol	Placebo		
Non	10 (55.6%)	2 (11.1%)	12 (33.3%)	0.018*
less than 50%	5 (27.8%)	11 (61.1%)	16 (44.4%)	
more than 50%	3 (16.7%)	5 (27.8%)	8 (22.2%)	
Total	18 (100%)	18 (100%)	18 (100%)	

Data as shown as n (%); * p <0 .05

Table S2 Read summary

Code	Group	input	filtered	denoisedF	denoisedR	merged	nonchim
H12	Healthy	45,420	39,364	38,814	39,197	38,384	38,030
H16	Healthy	26,423	22,950	22,750	22,797	22,458	22,264
H20	Healthy	35,686	30,801	30,520	30,597	30,082	29,452
H22	Healthy	43,077	38,144	37,963	38,029	37,674	37,074
H24	Healthy	11,152	9,205	9,146	9,158	9,077	9,077
H26	Healthy	23,922	20,212	19,936	19,984	19,542	19,493
H29	Healthy	68,397	59,110	58,109	58,614	56,554	52,729
H30	Healthy	43,975	37,572	36,562	37,126	35,370	32,529
H1	Healthy	31,218	26,621	26,354	26,398	25,963	25,882
H2	Healthy	27,419	23,909	23,626	23,739	23,287	23,263
H3	Healthy	26,469	23,318	23,076	23,186	22,909	22,876
H4	Healthy	38,937	34,034	33,799	33,914	33,463	32,993
H7	Healthy	44,557	36,014	35,588	35,793	35,072	34,699
H8	Healthy	42,792	36,943	36,527	36,743	35,738	34,882
H11	Healthy	49,569	40,177	39,398	39,788	38,230	35,361
T1	Oligonol_wk0	50,489	44,808	36,792	42,641	30,874	27,282
T27	Oligonol_wk0	50,840	44,673	39,075	42,891	28,339	20,829
T39	Oligonol_wk0	53,264	45,182	37,985	43,791	29,388	20,269
T28	Oligonol_wk0	50,437	44,309	38,570	42,542	29,159	21,261
T40	Oligonol_wk0	50,743	44,468	37,744	42,669	26,976	21,770
T17	Oligonol_wk0	56,134	49,260	40,026	46,326	31,230	25,873
T29	Oligonol_wk0	50,425	44,340	38,735	42,668	30,279	26,346
T19	Oligonol_wk0	43,756	38,102	32,704	36,605	24,000	20,386
T6	Oligonol_wk0	55,074	48,128	42,604	46,220	31,418	24,206
T21	Oligonol_wk0	50,803	44,571	39,183	42,030	28,692	23,391
T33	Oligonol_wk0	55,022	47,871	39,433	45,581	30,826	21,989
T22	Oligonol_wk0	54,159	46,026	39,376	44,313	29,400	22,692

T9	Oligonol_wk0	52,878	45,778	39,420	44,494	29,236	21,096
T11	Oligonol_wk0	51,699	45,322	34,399	42,609	25,915	19,798
T24	Oligonol_wk0	46,172	40,919	33,126	40,210	29,386	27,424
T36	Oligonol_wk0	51,078	44,822	39,640	43,198	31,396	27,528
T13	Oligonol_wk0	49,807	44,195	35,900	42,618	26,400	18,064
T37	Oligonol_wk0	51,597	45,077	37,792	43,752	29,101	21,983
T14	Oligonol_wk0	48,437	42,366	35,241	38,745	27,639	21,470
TA1	Oligonol_wk24	42,460	35,799	27,655	33,450	19,460	18,255
TA27	Oligonol_wk24	46,982	39,069	30,522	36,300	19,035	16,616
TA39	Oligonol_wk24	49,148	38,534	32,609	36,527	18,411	13,244
TA28	Oligonol_wk24	51,059	42,092	34,368	37,351	19,055	13,661
TA40	Oligonol_wk24	46,830	38,203	31,198	36,283	21,375	20,141
TA17	Oligonol_wk24	50,410	40,663	32,093	37,994	19,298	16,709
TA29	Oligonol_wk24	53,346	42,973	32,630	38,141	19,281	16,645
TA19	Oligonol_wk24	45,535	36,121	29,442	34,499	17,161	14,744
TA6	Oligonol_wk24	53,354	43,350	34,307	39,905	21,841	16,872
TA21	Oligonol_wk24	48,082	37,711	28,817	36,148	17,487	14,793
TA33	Oligonol_wk24	49,517	40,455	30,269	36,077	18,425	15,870
TA22	Oligonol_wk24	44,781	35,080	27,690	33,813	19,361	19,184
TA9	Oligonol_wk24	55,618	44,438	37,570	42,096	21,811	17,772
TA11	Oligonol_wk24	55,915	45,969	34,885	42,528	22,421	18,494
TA24	Oligonol_wk24	54,054	43,904	34,378	41,291	21,708	18,343
TA36	Oligonol_wk24	51,776	42,110	33,311	39,445	19,614	17,700
TA13	Oligonol_wk24	83,548	68,439	53,398	62,285	30,106	18,434
TA37	Oligonol_wk24	49,191	40,626	32,662	38,105	21,465	19,855
TA14	Oligonol_wk24	81,171	66,076	50,173	60,106	32,188	25,049
T15	Placebo_wk0	47,815	42,576	37,721	41,671	30,937	25,306
T2	Placebo_wk0	53,329	46,808	39,295	44,104	31,462	26,410
T16	Placebo_wk0	52,140	46,030	40,264	44,729	33,323	30,500
T3	Placebo_wk0	52,325	45,409	41,183	44,201	32,104	25,592
T4	Placebo_wk0	53,422	47,184	39,816	44,552	29,944	22,479
T18	Placebo_wk0	49,985	43,894	36,401	42,867	28,266	22,750
T30	Placebo_wk0	53,988	47,265	39,937	46,073	32,636	23,456
T5	Placebo_wk0	53,413	45,916	38,252	43,487	26,786	21,916
T31	Placebo_wk0	49,598	43,799	36,062	41,423	27,779	22,368
T20	Placebo_wk0	50,755	44,674	36,380	43,572	28,944	21,113
T32	Placebo_wk0	51,269	45,264	35,945	44,203	29,421	25,287
T7	Placebo_wk0	49,515	43,448	39,286	41,766	28,621	22,257
T8	Placebo_wk0	50,352	44,213	38,377	42,717	29,393	24,710
T34	Placebo_wk0	51,560	45,590	35,626	41,131	27,345	22,183
T23	Placebo_wk0	50,605	44,957	38,335	43,607	28,506	21,192
T35	Placebo_wk0	52,473	45,478	39,591	43,365	28,743	24,179
T25	Placebo_wk0	53,556	46,649	37,899	43,934	27,642	19,982
T26	Placebo_wk0	46,039	40,997	37,434	40,291	32,104	30,260
T38	Placebo_wk0	47,743	41,764	34,749	40,551	26,923	22,822
TA15	Placebo_wk24	48,962	40,385	33,028	38,390	18,979	12,845
TA2	Placebo_wk24	52,187	42,716	33,787	40,092	23,470	21,082
TA16	Placebo_wk24	51,850	42,071	33,628	39,335	21,771	18,779

TA3	Placebo_wk24	54,564	44,875	32,210	40,071	20,767	18,274
TA4	Placebo_wk24	53,323	41,366	33,513	39,547	20,866	17,313
TA18	Placebo_wk24	49,169	40,319	32,412	37,824	20,231	15,774
TA30	Placebo_wk24	48,958	40,019	32,429	36,480	18,899	13,295
TA5	Placebo_wk24	55,246	44,384	33,788	40,959	20,368	16,342
TA31	Placebo_wk24	46,827	38,129	29,907	35,745	18,345	13,876
TA20	Placebo_wk24	47,230	38,451	30,306	36,245	17,575	13,599
TA32	Placebo_wk24	50,671	41,244	32,789	38,851	18,512	14,959
TA7	Placebo_wk24	51,866	42,566	35,024	40,279	21,946	18,086
TA8	Placebo_wk24	54,884	45,026	34,944	42,269	20,874	16,672
TA34	Placebo_wk24	53,383	44,202	31,798	37,583	18,733	15,524
TA23	Placebo_wk24	49,364	40,762	32,648	38,437	21,431	17,378
TA35	Placebo_wk24	51,741	40,764	31,805	38,136	19,705	17,296
TA25	Placebo_wk24	41,323	34,449	27,908	32,675	16,966	13,880
TA26	Placebo_wk24	49,498	40,138	29,368	35,487	20,022	17,959
TA38	Placebo_wk24	50,755	41,405	31,612	38,838	18,672	15,995

Data as shown in average; wk0, week 0; wk24, week 24

Table S3 Post and pre-treatment changes (Δ) of relative abundance at family level in the oligonol and placebo groups

Top 20 at family level	ΔOligonol		ΔPlacebo		*p*−Value
	Mean	SD	Mean	SD	
f_Lachnospiraceae	−0.057	0.061	−0.051	0.059	0.942
f_Bacteroidaceae	0.037	0.073	0.047	0.062	0.204
f_Ruminococcaceae	−0.007	0.039	−0.039	0.051	0.034 *
f_Prevotellaceae	0.009	0.098	0.021	0.1	0.350
f_Bifidobacteriaceae	-0.004	0.043	0.007	0.028	0.204
f_Oscillospiraceae	0.005	0.016	−0.004	0.023	0.274
f_Enterobacteriaceae	0.011	0.029	0.01	0.046	0.672
f_Acidaminococcaceae	0.001	0.013	−0.003	0.02	0.589
f_Selenomonadaceae	−0.005	0.039	0.001	0.023	0.915
f_Coriobacteriaceae	−0.009	0.011	−0.006	0.023	0.988
f_Fusobacteriaceae	0.006	0.025	0.011	0.025	0.115
f_Sutterellaceae	0.004	0.009	0.003	0.012	0.737
f_Erysipelatoclostridiaceae	−0.008	0.014	0.006	0.021	0.024 *
f_Veillonellaceae	0.007	0.016	0.005	0.021	0.236
f_Rikenellaceae	0.004	0.015	0	0.013	0.630
f_Tannerellaceae	0.004	0.007	0	0.011	0.261
f_Erysipelotrichaceae	0.001	0.015	−0.003	0.011	0.184
f_Streptococcaceae	−0.002	0.013	0.005	0.028	0.559
f_Peptostreptococcaceae	−0.004	0.009	0.001	0.014	0.274
f_Succinivibrionaceae	0.003	0.012	0.005	0.016	0.408

Δ, Changes in the relative abundance of the gut microbiota between post- and pre-treatment. * $p < 0.05$.

Table S4 Patients with percent MRI-PDFF decreased ≥10% at week 24

**Sample ID**	**Group**	**BCoAT_wk0**	**BCoAT_wk24**
T31	%MRI-PDFF decreased (≥10%)_Placebo	0.001	0.010
T34	%MRI-PDFF decreased (≥10%)_Placebo	0.010	0.016
T35	%MRI-PDFF decreased (≥10%)_Placebo	0.023	0.007
T3	%MRI-PDFF decreased (≥10%)_Placebo	0.035	0.021
T4	%MRI-PDFF decreased (≥10%)_Placebo	0.003	0.017
T7	%MRI-PDFF decreased (>10%)_Placebo	0.021	0.010
T2	%MRI-PDFF decreased (≥10%)_Placebo	0.004	0.007
T18	%MRI-PDFF decreased (≥10%)_Placebo	0.037	0.004
T16	%MRI-PDFF decreased (≥10%)_Placebo	0.016	0.001
T15	%MRI-PDFF decreased (≥10%)_Placebo	0.011	0.017
T39	%MRI-PDFF decreased (≥10%)_Oligonol	0.004	0.016
T6	%MRI-PDFF decreased (≥10%)_Oligonol	0.016	0.055
T24	%MRI-PDFF decreased (≥10%)_Oligonol	0.022	0.081
T33	%MRI-PDFF decreased (≥10%)_Oligonol	0.016	0.018
T21	%MRI-PDFF decreased (≥10%)_Oligonol	0.007	0.072
T28	%MRI-PDFF decreased (≥10%)_Oligonol	0.028	0.005
T40	%MRI-PDFF decreased (≥10%)_Oligonol	0.035	0.015
T9	%MRI-PDFF decreased (≥10%)_Oligonol	0.024	0.020
T22	%MRI-PDFF decreased (≥10%)_Oligonol	0.017	0.019
T11	%MRI-PDFF decreased (≥10%)_Oligonol	0.025	0.021
T13	%MRI-PDFF decreased (≥10%)_Oligonol	0.004	0.007

MRI-PDFF, Magnetic Resonance Imaging Proton Density Fat Fraction; BCoAT, Butyryl-CoA: Acetate CoA-Transferase; wk0, week 0; wk24, week 24
